# Progress made in digitalizing antimicrobial resistance surveillance in a One Health approach in Kenya

**DOI:** 10.3389/fpubh.2024.1411962

**Published:** 2024-09-30

**Authors:** Veronicah M. Chuchu, Joseph Njung’e, Bridgit Muasa, Muchira Gathira, Geoffrey Olela, Kinuthia S. Bubi, Justus Ashaba, Susan Githii, Romona Ndanyi, Emmanuel Tanui, John Irungu Irungu, Allan Azegele, Rigveda Kadam, Cecilia Ferreyra

**Affiliations:** ^1^FIND, Geneva, Switzerland; ^2^National Veterinary Reference Laboratories, Directorate of Veterinary Services, Kabete, Kenya; ^3^National Antimicrobial Stewardship Interagency Committee (NASIC), Ministry of Health and the Ministry of Agriculture and Livestock Development, Nairobi, Kenya

**Keywords:** One Health, surveillance, digital health, interoperability, antimicrobial resistance

## Abstract

**Background:**

Antimicrobial resistance (AMR) is a major threat to global public health, affecting human and animal health, agriculture, food safety, and the environment. The control of AMR is often challenging, particularly when data are scanty or siloed in individual sectors. To develop evidence-based control policies for AMR, an electronic information system that integrates AMR data from various sectors, in a One Health approach, is critical.

**Methodology:**

Acknowledging the interconnectedness of AMR in humans, animals, and the environment and the need to assess the AMR burden using a One Health approach, Kenya’s National Antimicrobial Stewardship Interagency Committee (NASIC), with support from FIND, integrated human and animal health AMR data at the national AMR data repository and developed the One Health AMR Surveillance System (OHAMRS). The OHAMRS comprises two core digital components: interoperability middleware for integrating data from various sources and a DHIS2 web portal for the analysis and visualization of AMR surveillance data from the human and animal health sectors. These components are scalable for future inclusion of data from other One Health sectors, e.g., the environment, food/feed, and aquaculture sectors.

**Results:**

The OHAMRS has 42 dashboards that facilitate the presentation, interpretation, and dissemination of actionable information relating to AMR, including 17 dashboards for human and animal health priority pathogens and 8 for drug-resistance indicators. The priority pathogen dashboards provide visualization of antimicrobial susceptibility patterns, resistance and susceptibility trends, resistance tables, and geospatial susceptibility maps. Other dashboards include surveillance sites and specimen reports, data completeness, data reconciliation, sample testing workload, a One Health intersectoral dashboard, and other reporting tools for diverse stakeholders.

**Discussion:**

Digitalizing AMR surveillance through a One Health lens is pivotal to understand AMR prevalence and patterns across various sectors. The OHAMRS provides comprehensive data analysis and presentation, informing policymaking on AMR control. Digital tools such as the OHAMRS are vital in facilitating the availability of data and actionable information on AMR required to address the AMR crisis in Kenya.

## Background

Antimicrobial resistance (AMR) poses a significant challenge to global public health, placing a heavy financial burden on national economies, healthcare systems, and communities. AMR arises due to the indiscriminate use of antimicrobials and insufficient antimicrobial stewardship, both of which are modifiable factors (1). This issue threatens the effective prevention and treatment of the ever-increasing range of infections among humans and animals, directly impacting food safety and security, health expenditure, and human and animal morbidity and mortality (2, 3). In 2019, 1.27 million deaths occurring globally were directly attributable to bacterial resistance to antibiotics, with most of these deaths occurring in western sub-Saharan Africa at 27.3 deaths per 100,000 (4). Without action, it is estimated that by 2050 the global public health consequences and economic costs of AMR will result in 10 million human fatalities annually, a 3.8% decrease in the global gross domestic product (5) and 24 million impoverished people (6–8).

The close interactions within the animal-human-environment triad enable diverse and complex pathways for the spread of AMR which can also contribute to the emergence of new AMR. The quantity of antimicrobials used in animals is estimated to exceed that in humans, as they are utilized not only for therapeutic purposes but also for prophylaxis and as growth promoters. Furthermore, antimicrobials used in agriculture inevitably seep into surrounding soil and water ecosystems, ultimately reaching human populations and wildlife increasing the risk of AMR (9–11). Improving surveillance in all the sectors is critical for keeping track of new resistance patterns and assessing the effectiveness of both local, national, and the global containment and mitigation strategies for AMR (1).

In recognition of this growing threat, the World Health Organization (WHO) developed a global action plan on AMR to ensure countries have the capacity to combat it (12). There is a need for a multisectoral One Health approach for AMR and to prevent future pandemics. This has led to the formation of quadripartite agencies involving teams from WHO, the Food and Agriculture Organization of the United Nations (FAO), the World Organization for Animal Health (WOAH), and the United Nations Environment Programme (UNEP), who have jointly developed a strategy and a global plan of action on One Health aimed at, among other objectives, fostering concerted efforts aimed at combating the threat of AMR (9, 13).

Containing and controlling AMR demands coordinated and collaborative efforts within and between sectors and stakeholders (14). The strategic framework for collaboration on AMR developed by the quadripartite organizations acknowledges the need to strengthen surveillance of AMR and antimicrobial use (AMU) in a One Health approach and analyze data in an integrated manner (15). Such data will aid in properly describing the AMR burden and patterns of AMU to inform effective public health responses (2, 12).

Advocating for investment to address AMR is often a challenge because AMR data are scarce and what data are available are often siloed in individual sectors. This makes reliable assessments of the AMR burden problematic and hence difficult to provide strong evidence required to drive the research and policy agenda forward to fight AMR (16, 17). In Kenya, the Ministry of Health (MoH) and the Ministry of Agriculture and Livestock Development (MALD) established the multisectoral National Antimicrobial Stewardship Interagency Committee (NASIC) to coordinate efforts across sectors and stakeholders. NASIC developed a National Policy and Action Plan (NAP) to prevent and contain AMR. The NAP recognizes the need for multisectoral collaboration to increase the knowledge and evidence base for AMR mitigation actions through strengthening research and surveillance (5). Due to the interconnectedness of epidemiological pathways between humans, animals, and the environment, the NAP envisages the development of a national One Health surveillance system that can systematically collect and analyze AMR data (5).

At surveillance sites and at the national and global level, the use of information and communication technology (ICT) can greatly reduce errors and improve the efficiency of data collection, transmission, analysis, and use. The absence of electronic information systems including hospital information systems and laboratory information systems, and the deployment of non-interoperable electronic information systems have been identified as barriers to generating comprehensive AMR data that can be used to develop appropriate AMR policies and strategies (18). The interconnectedness of AMR in the human, animal, and environmental sectors require an electronic information system that employs a One Health approach and integrates AMR data from each of these sectors. However, there is a major shortage of such systems that can integrate AMR data from the human, animal, and environmental sectors (18).

Here, we describe the AMR surveillance system in Kenya and the progress made in digitalizing it by using a One Health approach. Specifically, we describe (1) the AMR surveillance system in Kenya, (2) the institutional capacity in the human and animal health sectors for the implementation of AMR surveillance, and (3) the capabilities of the digitalized One Health AMR Surveillance System (OHAMRS). We also highlight opportunities for further improvements that can build on the existing OHAMRS.

## The AMR surveillance system in Kenya

### Human AMR surveillance and information systems situation analysis

AMR surveillance in Kenya began in the late 1970s when the University of Nairobi and Kenyatta National Hospital started compiling and monitoring antibiotic susceptibility patterns in routine clinical isolates (19). Coordinated national AMR surveillance efforts, however, began in 2017 with the launch of the AMR NAP (5). Later, in 2018, the MoH launched the AMR Surveillance Strategy (2018–2022), with the primary goal of monitoring through laboratory-based surveillance the national burden of AMR among priority pathogens: *Escherichia coli*, *Klebsiella pneumoniae*, *Acinetobacter baumannii*, *Staphylococcus aureus*, *Streptococcus pneumoniae*, *Shigella* species, *Salmonella* species, and *Pseudomonas aeruginosa* (20). These priority pathogens were based on the WHO priority pathogen list and organisms of local priority. Among the selected priority organisms, *Escherichia coli*, *Klebsiella pneumoniae*, *Staphylococcus aureus*, *Streptococcus pneumoniae*, and *Pseudomonas aeruginosa* are reported to be responsible for 54.9% of deaths caused by 33 bacterial pathogens globally (21).

**Figure 1:**
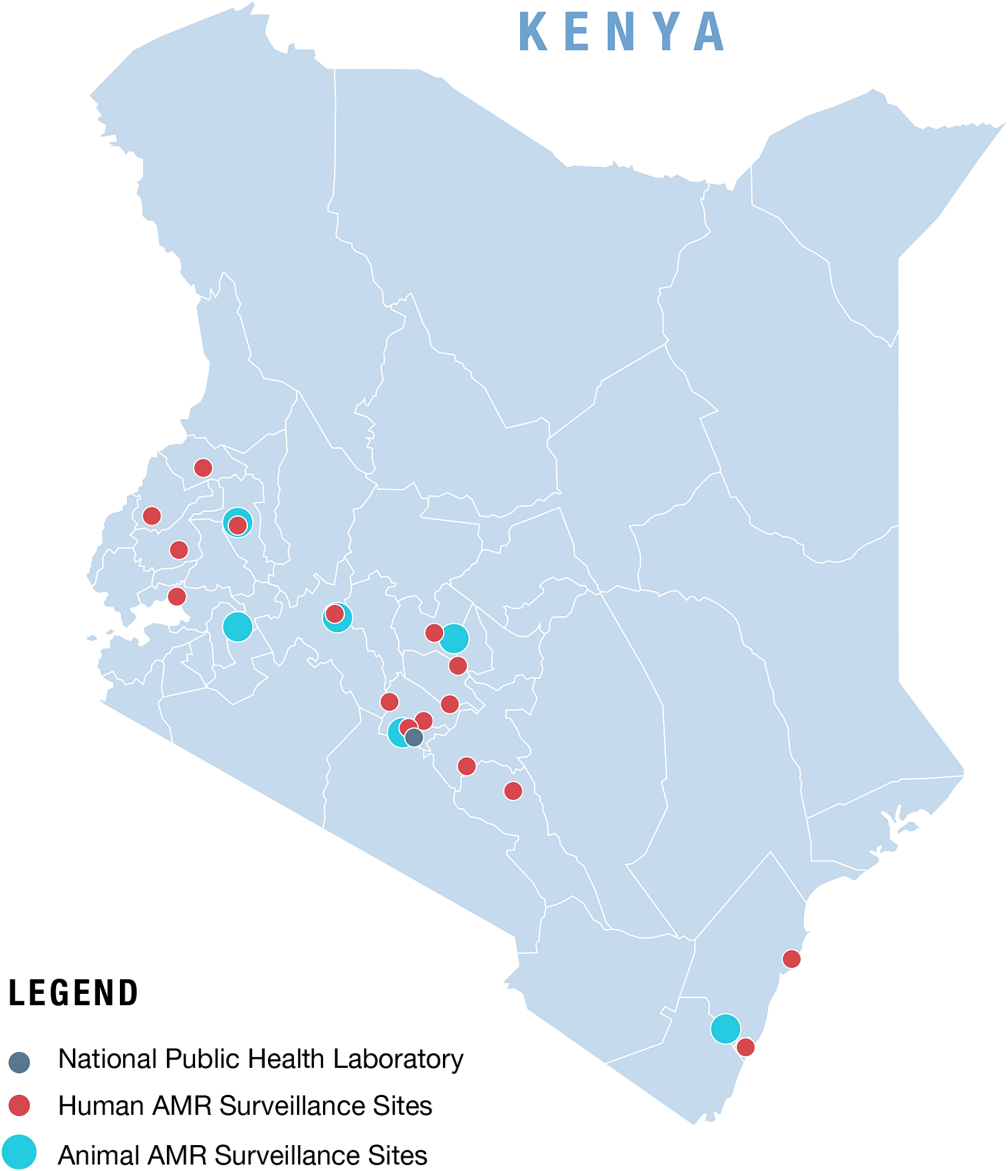
Map of Kenya showing the distribution of current AMR surveillance sites in the human and animal health sectors.

In 2016, two model sentinel surveillance hospital laboratories were enrolled as pilot sites. These two sites were initially assessed by NASIC with support from the US Centers for Disease Control and Prevention (US CDC) to identify their capacity to test and report AMR as per the national guidelines. The assessment revealed that although laboratory information management systems (LIMS) were in place in these laboratories, there was a need to update these systems to collect AMR data for reporting (22, 23). Furthermore, the data from these facilities were not readily available at the national level for analysis and feedback to the sites. Therefore, NASIC, through the National Public Health Laboratories (NPHL) ICT team, developed a national data repository referred to as the Central Data Warehouse (CDW) which archives the AMR data. The AMR data from the two sites, collected either through their LIMS or via the standard MoH Microsoft (MS) Excel-based data collection tool, were transmitted to and stored in the CDW. The sentinel sites submitted their first AMR data to the CDW in 2018. Later, the pilot was scaled up to include two additional laboratories. To date, the number of laboratories enrolled in the surveillance system has increased to 17 as shown in the map of Kenya (Figure 1), through the concerted efforts made by the national and county governments and with the support of partners (24). The sites submit AMR data from clinical samples for patients with suspected bacterial infections. NPHL maintains the CDW with the objective of compiling national AMR data for analysis, dissemination, and to inform national and county policies and practices, as well as for dissemination to the WHO Global Antimicrobial Resistance and Use Surveillance System (GLASS) (25).

Laboratories are enrolled in the surveillance system based on their baseline capacity to offer bacteriology culture services and commitment to routinely submit AMR data, conforming to the standard minimum set of data elements, to the CDW. Surveillance sites can report AMR data to the CDW in one of three ways: in near-real-time using an LIMS linked to the CDW, whereby data are transmitted directly to the CDW; on a weekly or monthly basis by sending WHONET data files to NPHL; or by collecting AMR data using the national standard MS-Excel tool developed by NPHL and sending the files on a monthly basis to NPHL to be uploaded to the CDW. Of the 17 surveillance sites enrolled in the surveillance system, 43% submit data using a LIMS, 38% via the MS-Excel template, and 19% via WHONET.

### Animal AMR surveillance and information systems situation analysis

In 2018, the MALD developed an AMR Surveillance Strategy for the agriculture sector, to guide the systematic collection and management of AMR data to inform policy decisions and improve clinical practice outcomes. The sector identified nine priority bacterial pathogens: *Campylobacter coli*, *Campylobacter jejuni*, *Enterococcus faecium*, *Enterococcus faecalis*, *Escherichia coli*, *Klebsiella pneumoniae*, *Pseudomonas aeruginosa*, *Salmonella* species, and *Staphylococcus aureus*. These priority pathogens were based on the WHO priority pathogen list and organisms of local priority.

Kenya has seven National Veterinary Laboratories (NVLs), with six of them conducting both passive and active AMR surveillance (the latter involves healthy poultry being sampled to detect the presence of antibiotic-resistant bacteria) (Figure 1). Until 2018, when an MS-Excel data collection tool was introduced, these facilities had been using paper-based tools for data collection and reporting. The use of a LIMS began in 2017, when one of the laboratories, the Central Veterinary Laboratories (CVL), acquired a LIMS known as Sistema Laboratorio (SILAB), designed for use in Africa and supported by the UN FAO. By 2021, all other NVLs had commissioned and installed SILAB. Also in 2021, a module specifically designed to collect AMR data was added to the SILAB system to collect AMR data variables, including laboratory ID, sample ID, reason for sampling, type of sample collected, animal species, test type, origin of sample (county, subcounty and ward), sampling date, isolation date, test date, report generation date, bacterial culture results, antimicrobial susceptibility test (AST) method, and AST results.

### Reporting AMR surveillance data to WHO GLASS

In 2017, Kenya enrolled in WHO GLASS for the submission of AMR data. In 2021, NASIC held an AMR technical working group (TWG) meeting to review and validate data from the CDW for submission to GLASS for the 2020 AMR data call. During this meeting, the TWG gave recommendations relating to strengthening the AMR data collection tools and capturing of key AMR variables, defined an appropriate reporting format for organisms, and streamlined “drug–bug” combination reporting and organism–specimen type/source reporting. In the 2021 and 2022 data calls, Kenya submitted data from 6 and 16 surveillance sites, respectively.

### Establishment of the One Health AMR surveillance system

The NAP objective to strengthen Kenya’s AMR surveillance system requires high-quality surveillance data to support evidence-based decision-making, the identification of priority areas for action, and to communicate the importance and impact of AMR to stakeholders and decision makers (5). The development of a national OHAMRS is one activity that contributes to this effort.

**Figure 2:**
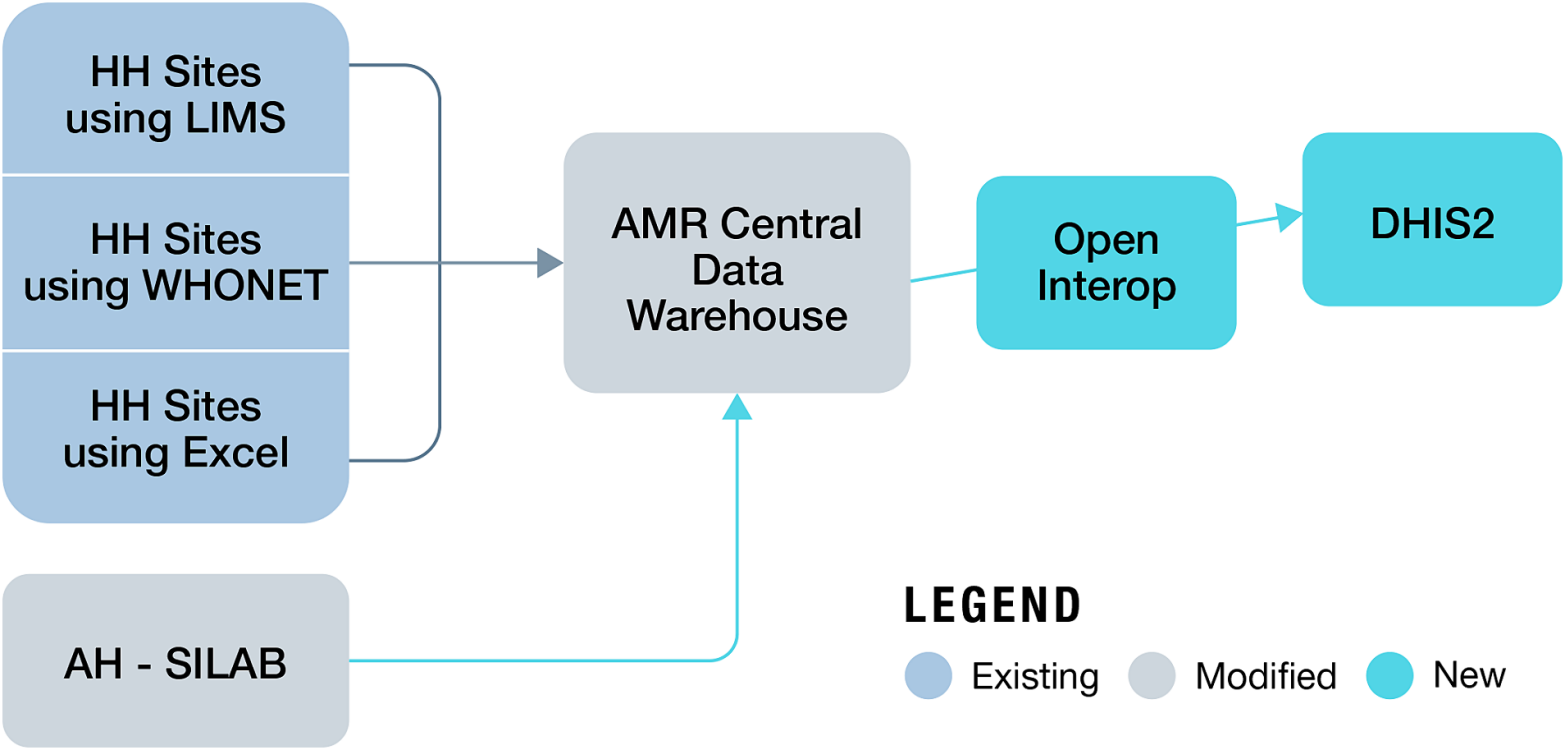
Data flow from human and animal surveillance sites to the One Health AMR Surveillance System (AH, animal health; HH, human health).

Building on the immense progress made in increasing the institutional capacity in AMR surveillance by the MoH and MALD, NASIC in collaboration with FIND, began developing the OHAMRS to collate AMR data from the human and animal health sectors in a single digital platform. FIND partnered with eSHIFT and Software for Health Foundation to design OHAMRS for Kenya based on a pilot implementation in Zambia in 2019–2020. This platform comprises open-source, middleware interoperability software, known as Open Interop (26), and District Health Information Software 2 (DHIS2), a popular, free, and open-source data analysis and visualization software tool currently in use in more than 73 countries worldwide. The CDW is connected to OHAMRS portal via Open Interop, allowing AMR data from the human and animal health sectors to be analyzed and visualized using DHIS2 (Figure 2). The visualization dashboards automatically update on a near-real-time basis as new data are submitted to the CDW. The platform can collect data, warehousing, visualization, and analytic live data in real time (27, 28). DHIS2 is used in many countries for the collection and reporting of health data. This has improved data use at both facility and national levels, helping to support decision-making (29, 30). DHIS2 has a vibrant user community, which makes it very appropriate for One Health AMR surveillance in Kenya.

### Implementation of the One Health AMR surveillance system

The implementation of OHAMRS was a collaborative effort coordinated by NASIC and led by FIND with partnership between eSHIFT, Software for Health Foundation, and SILAB Africa team. We delineated the scope and requirements of the surveillance system by conducting regular engagement sessions that involved relevant stakeholders. Throughout the project we organized numerous showcases to ensure that user needs, especially those related to data analysis and visualization using the dashboards, were thoroughly integrated. Recognizing that the sustained implementation and effective utilization of the OHAMRS hinged on the system’s usability and the availability of skilled personnel, we provided comprehensive training to stakeholders and users at both the national and surveillance site levels. This training encompassed system manipulation and the creation of new dashboards, charts, maps, and tables. Also, ICT professionals responsible for maintaining the system received training in various technical aspects, including system backup and recovery, data analytics, DHIS2 configuration, and data loading procedures. Ultimately, the success of the OHAMRS will be gauged by the extent to which the target audience leverages the data collected for evidence-based decision-making at both facility and national levels.

### Utility of the One Health AMR surveillance system

The OHAMRS features a range of customizable dashboards that offer visualizations of data from both the human and animal health sectors. These dashboards address key concerns such as trends and patterns in AMR resistance and testing workload. Additionally, the platform includes a GLASS reporting module, streamlining the computation and preparation of annual reports for submission to the WHO GLASS.

**Figure 3:**
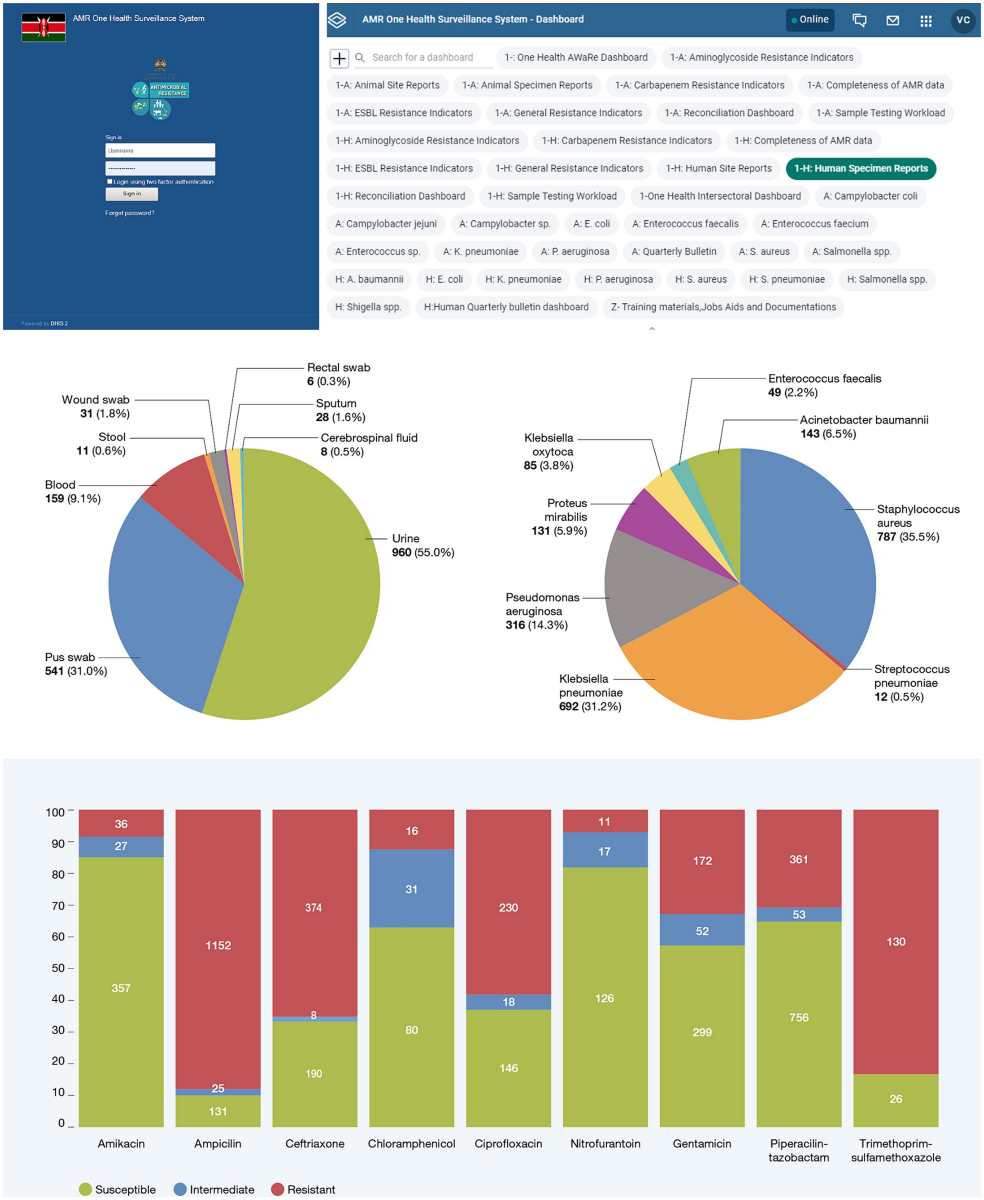
Screenshots of some of the One Health AMR Surveillance System dashboards.

The OHAMRS simplifies data analysis processes and promotes widespread utilization of AMR data at the national, county, and surveillance site levels. This will facilitate the early detection of resistant pathogens, informing policy decisions about interventions to combat AMR. Currently, the OHAMRS boasts 42 dashboards (Figure 3), including 17 that are dedicated to human and animal health AMR priority pathogens. The remaining dashboards cover surveillance site and specimen reports, data completeness, data reconciliation, sample testing workload, One Health intersectoral analysis, One Health AWaRe (Access, Watch and Reserve) classification, and quarterly bulletins (Supplementary Table 1).

To examine and depict trends in antimicrobial resistance within Kenya, the OHAMRS features priority pathogen dashboards. These dashboards include charts illustrating the types of samples from which organisms were isolated; susceptibility patterns for these organisms, presented via charts and tables; susceptibility patterns for organisms isolated from the predominant sample types; resistance trends observed over specific timeframes; and geospatial maps illustrating drug-resistance patterns over time. Additionally, to visualize antibiogram reports for each organism, tables displaying susceptibility and resistance percentages, along with numerators and denominators, have been incorporated in the system. The analyses derived from these priority pathogen dashboards offer crucial visual insights into resistance trends and patterns within both the human and animal sectors in Kenya. This information serves as valuable evidence to inform infection prevention and control measures, diagnostic and antimicrobial stewardship efforts, and the identification of research areas that could contribute to effective AMR control.

The surveillance site report dashboards include tables displaying various metrics, such as the frequencies and types of samples submitted by each facility over time, the frequencies of pathogens isolated per facility, the number of antimicrobials tested per facility, the methods employed for testing, the number of cultures that have had/not had ASTs, the number of specimens submitted per animal species, and the number of antibiotics tested for each organism.

These dashboards serve several important purposes:

1. They offer a comprehensive overview of organisms isolated beyond the priority pathogens. This information is critical for assessing changes in the rates at which these pathogens are isolated, to inform the necessary actions to be taken.
2. They enable the tracking of surveillance site workloads, helping to identify increased demand for diagnostic capacity and sites that may require clinician awareness campaigns; this tracking also allows the triggering of follow-up in cases where there may be decreased diagnostic usage at site level.
3. They provide insights into the profile of antibiotics used for ASTs within the facilities.
4. They provide data on samples that have been cultured and which cultures have undergone AST.

To assess the quality of data submitted by surveillance sites and evaluate their workload, the specimen report dashboard presents the distribution of sample types categorized by surveillance facility and the types of samples submitted over a specified timeframe. Given that blood, urine, and pus swabs constitute the majority of samples submitted in human health facilities, while milk and cloacal swabs are the most common sample types in animal health laboratories, the dashboard also highlights the proportions of organisms isolated from these sample types.

The data completeness dashboard provides information on the total number of samples tested within a given period. It evaluates data completeness in relation to specimen type, test method, sampling purpose, animal species, age, sex, diagnosis, specimen type, ward of admission, and identified organisms. These dashboards are valuable tools that can assist surveillance sites in assessing the quality of their data.

To track surveillance site workload we developed the sample testing workload dashboard, which presents total samples tested per facility over a given time period, types of samples tested per facility, cultures conducted, organisms identified in cultures conducted without proceeding to an AST, organisms identified in cultures proceeding to an AST, trends in sample workload over time by facility and county, and trends in cultures with or without an AST over a given time-frame.

Multidrug-resistant infections require careful attention due to the limited availability of antimicrobials available to treat these infections, their association with prolonged hospital stays, increased treatment costs, and increased patient mortality. To monitor multidrug-resistant organisms (MDROs), we developed drug-resistance indicator dashboards to visualize patterns for methicillin-resistant *Staphylococcus aureus*, extended-spectrum beta-lactamases, vancomycin-resistant enterococci, vancomycin-resistant *S. aureus*, carbapenem-resistant Enterobacteriaceae, and aminoglycoside-resistant gram-negative bacilli. The availability of these data will provide valuable insights into the presence of MDROs, informing interventions aimed at reducing MDRO infections and transmission.

To compare all the data submitted to the OHAMRS with the raw data held in the CDW, we created a reconciliation dashboard to present summary tables of the data held in the OHAMRS. These tables encompass total data submitted to the OHAMRS over time, total data submitted stratified by patient age and sex, surveillance site, sample type, and cultures with or without AST and their corresponding susceptibility patterns.

The WHO requires countries to report their AMR data via GLASS. The process includes converting aggregate national AMR data into simple text-based data file formats, including RIS files, with AST results and sample files that contain the number of patients from which specimens have been taken. OHAMRS has a GLASS module that generates WHO RIS and sample files which can be downloaded for submission to WHO GLASS.

## Conclusion

Kenya has made tremendous strides in digitalizing its AMR surveillance. The availability of the OHAMRS will aids in the detection, reporting, and characterization of resistant pathogens which is critical in developing evidence-based policies for control of resistant pathogens. However, there is a need to continue building a robust surveillance system to incorporate antimicrobial consumption and use data. The availability of these data, together with AMR data, will be used to identify associations between AMR and antimicrobial consumption, which is essential for informing and evaluating appropriate public health actions. Furthermore, there is a need to improve the representativeness of AMR data in the country to allow comparisons among settings and gain more accurate estimates of the AMR burden in Kenya, by increasing the number of AMR surveillance sites. As there are still significant data gaps to support the fight against AMR, it is critical to prioritize funding in building laboratory infrastructure and strengthening health system capacity in Kenya to increase the number of AMR surveillance sites.

In addition, the environmental sector is increasingly recognized as contributing to the development and spread of AMR (31, 32). To better understand patterns of AMR in this sector, evidence is required. This could be achieved by incorporating data from the environmental sector into the OHAMRS.

Robust surveillance of antimicrobial resistant organisms across the One Health sectors is needed for effective evidence-based policy, stewardship, and control measures that take into account the complex interconnections among humans, animals, and the environment. The OHAMRS creates an opportunity for the visualization of these data to inform policy and decision-making in relation to the control of AMR.

## References

[1] WHO. Global antimicrobial resistance and use surveillance system (GLASS) report. (2020). Available at: https://www.who.int/publications/i/item/9789240005587 (Accessed April 23, 2023).

[2] WHO. Antimicrobial resistance: global report on surveillance. WHO Libr Cat Data Antimicrob. (2014) 30:619–35.

[3] Prestinaci F Pezzotti P Pantosti A. Antimicrobial resistance: a global multifaceted phenomenon. Pathog Glob Health. (2015) 109:309–18. doi: 10.1179/2047773215Y.0000000030, PMID: 26343252 PMC4768623

[4] Antimicrobial Resistance Collaborators. Global burden of bacterial antimicrobial resistance in 2019: a systematic analysis. Lancet. (2022) 399:629–55. doi: 10.1016/S0140-6736(21)02724-0 35065702 PMC8841637

[5] Government of Kenya. Republic of Kenya National Policy on prevention and containment of antimicrobial resistance. (2017). Available at: www.health.go.ke

[6] O’Neill J. Tackling drug-resistant infections globally: final report and recommendations. London: Review on Antimicrobial Resistance. (2016).

[7] Neill JO. Antimicrobial resistance: tackling a crisis for the health and wealth of nations. London: Review on Antimicrobial Resistance. (2014).

[8] World Bank. Drug-resistant infections: a threat to our economic future, vol. 2 Washington, D.C. USA: World Bank Report (2016).

[9] WHO Regional Office for Europe. Multisectoral action to tackle antimicrobial resistance. Regional Office for Europe, Denmark: World Health Organization (WHO). (2019).

[10] Ikhimiukor OO Odih EE Donado-Godoy P Okeke IN. A bottom-up view of antimicrobial resistance transmission in developing countries. Nat Microbiol. (2022) 7:757–65. doi: 10.1038/s41564-022-01124-w 35637328

[11] Velazquez-Meza ME Galarde-López M Carrillo-Quiróz B Alpuche-Aranda CM. Antimicrobial resistance: one health approach. Vet World. (2022) 15:743–9. doi: 10.14202/vetworld.2022.743-749, PMID: 35497962 PMC9047147

[12] WHO. Global action plan on antimicrobial resistance. WHO Libr Cat Data Antimicrob. (2015)

[13] FAO, UNEP, WHO, WOAH. Global plan of action on one health. Towards a more comprehensive One Health, approach to global health threats at the human-animal-environment interface. (2022). doi: 10.4060/cc2289en

[14] WHO. Tackling antimicrobial resistance together. Multisectoral coordination. (2018). Available at: https://www.who.int/publications/i/item/tackling-antimicrobial-resistance-together-working-paper-1.0-multisectoral-coordination (Accessed August 16, 2024).

[15] WHO, FAO, OIE, UNEP. Strategic framework for collaboration on antimicrobial resistance – together for one health. (2022). Available at: www.who.int

[16] Hay SI Rao PC Dolecek C Day NPJ Stergachis A Lopez AD. Measuring and mapping the global burden of antimicrobial resistance. BMC Med. (2018) 16:78. doi: 10.1186/s12916-018-1073-z, PMID: 29860943 PMC5985573

[17] Dunachie SJ Day NPJ Dolecek C. The challenges of estimating the human global burden of disease of antimicrobial resistant bacteria. Curr Opin Microbiol. (2020) 57:95–101. doi: 10.1016/j.mib.2020.09.013, PMID: 33147565 PMC7763986

[18] Oberin M Badger S Faverjon C Cameron A Bannister-Tyrrell M. Electronic information systems for one health surveillance of antimicrobial resistance: a systematic scoping review. BMJ Glob Heal. (2022) 7:1–10. doi: 10.1136/bmjgh-2021-007388 PMC8728452 34983786

[19] Revathi G Mailu C. Kenya’s perspective on antibiotic resistance. Int J Infect Dis. (2016) 45:10. doi: 10.1016/j.ijid.2016.02.054

[20] Ministry of Health. National antimicrobial resistance surveillance strategy. Ministry of Health Kenya. (2018).

[21] Antimicrobial Resistance Collaborators. Global mortality associated with 33 bacterial pathogens in 2019: a systematic analysis for the global burden of disease study 2019. Lancet. (2022) 400:2221–48. doi: 10.1016/S0140-6736(22)02185-7 36423648 PMC9763654

[22] CDC. Strengthening antimicrobial resistance surveillance. Glob Health. (2018). Available at: https://archive.cdc.gov/www_cdc_gov/globalhealth/countries/kenya/blog/strengthening-antimicrobial-resistance-surveillance.html (Accessed August 8, 2024).

[23] CDC. Tracking antibiotic resistance in Kenya and Senegal. CDC. (2019). Available at: https://archive.cdc.gov/www_cdc_gov/drugresistance/solutions-initiative/stories/surveillance-in-Kenya-Senegal.html (Accessed August 8, 2024).

[24] National Antimicrobial Stewardship Interagency Committee Secretariat. National antimicrobial resistance surveillance report. Kenya: Ministry of Health/Ministry of Agriculture and Livestock Development. (2022)

[25] World Health Organization. WHO global antimicrobial resistance and use surveillance system (GLASS). Available at: https://www.who.int/initiatives/glass (Accessed August 8, 2024).

[26] The Software for Health Foundation. Open Interop. Available at: softwareforhealth.org.

[27] Moirongo RM Aglanu LM Lamshöft M Adero BO Yator S Anyona S. Laboratory-based surveillance of antimicrobial resistance in regions of Kenya: an assessment of capacities, practices, and barriers by means of multi-facility survey. Front Public Heal. (2022) 10:1003178. doi: 10.3389/fpubh.2022.1003178 PMC9742437 36518572

[28] DHIS2. District health information system. Available at: https://dhis2.org/

[29] Manya A Braa J Øverland L Titlestad O Mumo J Nzioka C. National roll out of district health information software (DHIS 2) in Kenya, 2011–central server and cloud based infrastructure. IST Africa. (2012) 2012:1–9.

[30] Karuri J Waiganjo P Orwa D Manya A. DHIS2: the tool to improve health data demand and use in Kenya. J Health Inform Dev Ctries. (2014) 8:38–60.

[31] Larsson DGJ Flach CF. Antibiotic resistance in the environment. Nat Rev Microbiol. (2022) 20:257–69. doi: 10.1038/s41579-021-00649-x, PMID: 34737424 PMC8567979

[32] Singer AC Shaw H Rhodes V Hart A. Review of antimicrobial resistance in the environment and its relevance to environmental regulators. Front Microbiol. (2016) 7:1728. doi: 10.3389/fmicb.2016.01728 27847505 PMC5088501

