# Supplementary material for: Progress made in digitalizing antimicrobial resistance surveillance in a One Health approach in Kenya
Source: Front Public Health. 2024 Sep 30;12:1411962. doi: 10.3389/fpubh.2024.1411962 (PMC11491712; doi:10.3389/fpubh.2024.1411962)
Supplement: Supplementary file 1 [file Table_1.docx]

**Supplementary Tables**

**Supplementary Table 1: Description of the OHAMRS dashboards**

|  | **Dashboard name** | **Dashboard description** |
| --- | --- | --- |
| 1. | Human priority pathogens | There are eight different dashboards, for *Acinetobacter baumannii,* *Escherichia coli, Klebsiella pneumoniae*, *Pseudomonas aeruginosa, Staphylococcus aureus*, *S*. *pneumonia*, *Salmonella* species, and *Shigella* species. |
| 2. | Animal priority pathogens | There are 11 different dashboards, for *Campylobacter coli*, *Campylobacter jejuni*, *Campylobacter* species, *Escherichia coli*, *Enterococcus faecalis*, *Enterococcus faecium*, *Enterococcus* species, *Pseudomonas aeruginosa*, *Staphylococcus aureus*, *Klebsiella pneumoniae*, and *Salmonella* species. |
| 3. | One Health intersectoral dashboard | This dashboard provides a comparison between Extended-spectrum beta-lactamases (ESBL) and Methicillin-resistant *Staphylococcus aureus* (MRSA) indicators between the human and animal health sectors. It also compares resistance and susceptibility for human and animal *Escherichia coli*, *Staphylococcus aureus*, and *Pseudomonas aeruginosa*. |
| 4. | One Health AWaRe classification dashboard | This dashboard has charts showing pathogens and their resistance to antibiotics as classified by WHO to Access, Watch and Reserve (AWaRe). |
| 5. | Resistance indicator dashboard for human and animal pathogens | |
|  | Methicillin-resistant *Staphylococcus aureus* (MRSA) | This dashboard presents a count (and percentage) of *Staphylococcus aureus* resistant to oxacillin and/or cefoxitin divided by the count of *Staphylococcus aureus* tested for oxacillin and/or cefoxitin. |
|  | Extended-spectrum beta-lactamases (ESBLs) | Presents a count (and percentage) of *Escherichia coli* and/or *Klebsiella* and/or *Proteus* and/or *Salmonella* and/or *Enterobacter* and/or *Citrobacter* resistant to ceftriaxone and/or ceftazidime and/or cefotaxime divided by a count of *Escherichia coli* and/or *Klebsiella* and/or *Proteus* and/or *Salmonella* and/or *Enterobacter* and/or *Citrobacter* tested for ceftriaxone and/or ceftazidime and/or cefotaxime. The dashboard also shows the resistance of each organism to the above antibiotics. |
|  | Vancomycin-resistant enterococci (VRE) | Presents a count of enterococci resistant to vancomycin divided by a count of enterococci tested for vancomycin. |
|  | Vancomycin-resistant *S*. *aureus* | Presents a count of *Staphylococcus* *aureus* resistant to vancomycin divided by a count of *Staphylococcus* *aureus* tested for vancomycin. |
|  | Carbapenem-resistant Enterobacteriaceae (CRE) | Presents a count of *Escherichia coli* and/or *Klebsiella* and/or *Proteus* and/or *Salmonella* and/or *Enterobacter* and/or *Citrobacter* resistant to meropenem and/or imipenem and/or ertapenem divided by *Escherichia coli* and/or *Klebsiella* and/or *Proteus* and/or *Salmonella* and/or *Enterobacter* and/or *Citrobacter* tested for meropenem and/or imipenem and/or ertapenem.  The dashboard also shows the resistance of each organism to the above antibiotics. |
|  | Aminoglycoside-resistant gram-negative bacilli | Presents a count (and percentage) of *Escherichia coli* and/or *Klebsiella* and/or *Proteus* and/or *Salmonella* and/or *Enterobacter* and/or *Citrobacter* and/or *Pseudomonas* and/or *Acinetobacter* resistant to amikacin and/or gentamicin divided by *Escherichia coli* and/or *Klebsiella* and/or *Proteus* and/or *Salmonella* and/or *Enterobacter* and/or *Citrobacter* and/or *Pseudomonas* and/or *Acinetobacter* tested for amikacin and/or gentamicin.  The dashboard also shows the resistance of each organism to the above antibiotics. |
| 6. | Completeness of AMR data | Two dashboards representing data from the animal and human health sectors. Each dashboard highlights the quality of data by plotting the completeness of data variables submitted by the surveillance sites. |
| 7. | Sample testing workload | The two dashboards for human and animal health track the workload of the surveillance sites. This includes the types of samples tested per facility, cultures conducted, organisms identified in cultures conducted without proceeding to AST, organisms identified in cultures proceeding to AST, trends of sample workload over time by facility and county, and trends of cultures with and without AST over a given time period. |
| 8. | Reconciliation dashboard | Two dashboards, representing human and animal health, present summary tables of the data available in the OHAMRS. These include total data submitted stratified by patient age and sex, surveillance site, sample type, and cultures with or without AST and their susceptibility patterns. |
| 9. | Surveillance site reports | These two dashboards represent human and animal health facilities. The dashboards include data stratified by surveillance sites in the form of frequency tables on sample types submitted over time, frequency of pathogens isolated per facility, number of antimicrobials tested per facility, test methods, number of cultures with and without AST, specimens submitted per animal species, and the number of antibiotics each organism is tested against. |
| 10. | Specimen report | This dashboard displays sample type distribution stratified by surveillance facility and sample types submitted over a given time period. As blood, urine, and pus swabs comprise most of the samples submitted in human health facilities, and milk and cloacal swabs predominate in animal health laboratories, the dashboard shows the proportions of organisms isolated from these samples. |
